# Operational research as implementation science: definitions, challenges and research priorities

**DOI:** 10.1186/s13012-016-0444-0

**Published:** 2016-06-06

**Authors:** Thomas Monks

**Affiliations:** NIHR CLAHRC Wessex, Faculty of Health Sciences, University of Southampton, Southampton, UK

## Abstract

**Background:**

Operational research (OR) is the discipline of using models, either quantitative or qualitative, to aid decision-making in complex implementation problems. The methods of OR have been used in healthcare since the 1950s in diverse areas such as emergency medicine and the interface between acute and community care; hospital performance; scheduling and management of patient home visits; scheduling of patient appointments; and many other complex implementation problems of an operational or logistical nature.

**Discussion:**

To date, there has been limited debate about the role that operational research should take within implementation science. I detail three such roles for OR all grounded in upfront system thinking: structuring implementation problems, prospective evaluation of improvement interventions, and strategic reconfiguration. Case studies from mental health, emergency medicine, and stroke care are used to illustrate each role. I then describe the challenges for applied OR within implementation science at the organisational, interventional, and disciplinary levels. Two key challenges include the difficulty faced in achieving a position of mutual understanding between implementation scientists and research users and a stark lack of evaluation of OR interventions. To address these challenges, I propose a research agenda to evaluate applied OR through the lens of implementation science, the liberation of OR from the specialist research and consultancy environment, and co-design of models with service users.

**Summary:**

Operational research is a mature discipline that has developed a significant volume of methodology to improve health services. OR offers implementation scientists the opportunity to do more upfront system thinking before committing resources or taking risks. OR has three roles within implementation science: structuring an implementation problem, prospective evaluation of implementation problems, and a tool for strategic reconfiguration of health services. Challenges facing OR as implementation science include limited evidence and evaluation of impact, limited service user involvement, a lack of managerial awareness, effective communication between research users and OR modellers, and availability of healthcare data. To progress the science, a focus is needed in three key areas: evaluation of OR interventions, embedding the knowledge of OR in health services, and educating OR modellers about the aims and benefits of service user involvement.

## Background

Operational research (OR) is the discipline of using models, either quantitative or qualitative, to aid decision-making in complex problems [1]. The practice of applied healthcare OR distinguishes itself from other model-based disciplines such as health economics as it is action research based where operational researchers participate collaboratively with those that work in or use the system to define, develop, and find ways to sustain solutions to live implementation problems [2]. The methods of OR have been used in healthcare since the 1950s [3] to analyse implementation problems in diverse areas such as emergency departments [4–6] and management policies for ambulance fleet [7]; acute stroke care [8–11], outpatient clinic waiting times [12], and locations [13]; cardiac surgery capacity planning [14]; the interface between acute and community care [15]; hospital performance [16]; scheduling and routing of nurse visits [17]; scheduling of patient appointments [18]; and many other complex implementation problems of an operational or logistical nature.

Implementation science is the study of methods to increase the uptake of research findings in healthcare [19]. Given the volume of OR research in healthcare implementation problems, it is remarkable that limited discussion of the discipline has occurred within the implementation science literature. A rare example of debate is given by Atkinson and colleagues [20] who introduce the notion of system science approaches for use in public health policy decisions. Their argument focused on two modelling methods, system dynamics and agent-based simulation, and the potential benefits they bring for disinvestment decisions in public health. To complement and extend this debate, I define the overlap between implementation science and OR. I have focused on the upfront role that OR takes when used as an implementation science tool. Although some detail of method is given, the full breath of OR is beyond the scope of this article; a detailed overview of all the methods can be found elsewhere [21]. I describe three roles for OR within implementation science: structuring an implementation problem, prospective evaluation of an intervention, and strategic reconfiguration of services. For each role, I provide a case study to illustrate the concepts described. I then describe the challenges for OR within implementation science at the organisational, interventional, and disciplinary levels. Given these challenges, I derive a research agenda for implementation science and OR.

## Discussion

### OR to structure an implementation problem

The first role for OR in implementation science is to provide a mechanism for structuring an implementation problem. Within OR, *problem structuring methods* provide participatory modelling approaches to support stakeholders in addressing problems of high complexity and uncertainty [22]. These complex situations are often poorly defined and contain multiple actors with multiple perspectives and conflicting interests [23]. As such, they are unsuitable for quantitative approaches. Problem structuring methods aim to develop models that enable stakeholders to reach a shared understanding of their problem situation and commit to action(s) that resolve it [23]. Approaches might serve as a way to clearly define objectives for a quantitative modelling study [24], systematically identify the areas to intervene within a system [25], or may be an intervention to improve a system in its own right.

#### A case example—understanding patient flow in the mental health system

A mental health service provider in the UK provided treatment to patients via several specialist workforces. Here, I focus on two: psychology and psychiatric talking therapies (PPT) and recovering independent life (RIL) teams. Waiting times to begin treatment under these services were high (e.g. for RIL team median = 55 days, inter-quartile range = 40–95 days), and treatment could last many years once it had begun. The trust’s management team were eager to implement new procedures to help staff manage case load and hence reduce waiting times to prevent service users, here defined as patients, their families, and carers, from entering a crisis state due to diminishing health without treatment. Management believed that reasons for delays were more complex than lack of staff, but the exact details were unclear and there was much disagreement between the senior management. The implementation science intervention I detail was conducted as an OR problem structuring exercise.

##### Methods

**Fig. 1:**
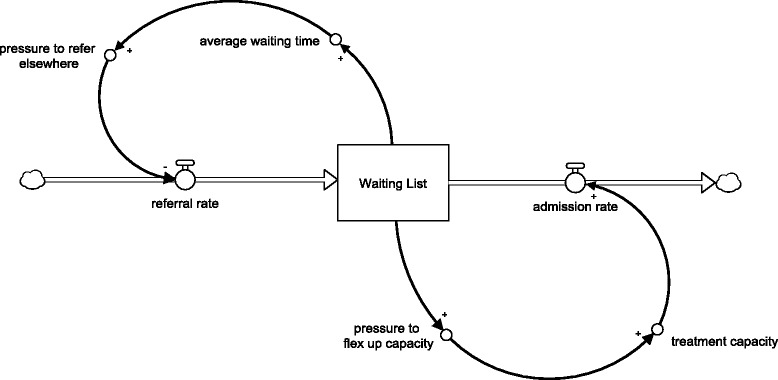
Example system thinking for a waiting list—stock and flow notation. Notation guide. *Rectangles* represent stocks which are acculations of quantity of interest; *Pipes with valves* represent flows which feed or deplete stocks; *arrows* represent how one aspect of a system positively or negatively influences another

A system dynamics (SD) model was constructed to aid management target their interventions. SD is a subset of system thinking—the process of understanding how things within a system influence one another within the whole. SD models can be either qualitative or quantitative. In this case, a purely qualitative model was created. Figure 1 illustrates stock and flow notation that is commonly used in SD. The example is the concept of a simple waiting list for a (generic) treatment. It can be explained as follows. General practitioners (GPs) refer service users to a waiting list at an average daily rate, while specialist clinicians treat according to how much daily treatment capacity they have. The variable *waiting list* is represented as a rectangular stock: an accumulation of patients. The waiting list stock is either depleted or fed by rate variables, referring and treating, represented as flows (pipes with valves) entering and leaving the stock. Figure 1 also contains two feedback loops that are illustrated by the curved lines. The first loop is related to the GP reluctance to refer to a service with a long waiting time. As the waiting list for a service increases in number, so does the average waiting time of service users and so does the pressure for GPs to consider an alternative service (lowering the daily referral rate). The second loop is related to specialist clinicians reacting to long waiting lists by creating a small amount of additional treatment capacity and increasing admission rates.

A preliminary version of the SD model was created using a series of interviews with clinicians and managers from the three services. This was followed by a group model building workshop that involved all senior management. Group model building is a structured process that aims to create a shared mental model of a problem [26]. The workshop began with a nominal group exercise. The group were asked to individually write down what they believed were the key factors that affected patient waiting times. The group were specifically asked to focus on strategic issues as opposed to detailed process-based problems. After all individual results had been shared, the group were asked to (i) hypothesise how these factors influenced each other and (ii) propose any missing variables that may mediate influence. For example, available treatment capacity is reduced by non-clinical workload. Non-clinical workload is increased by several other factors (discussed below in results) and so on.

##### Results

**Fig. 2:**
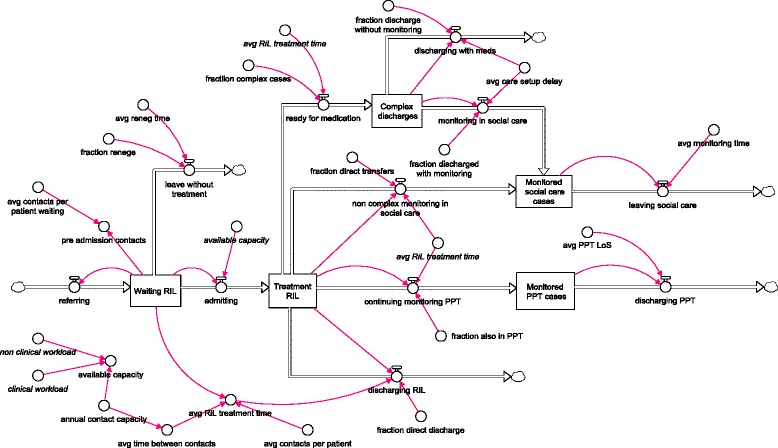
A simplified version of the RIL team patient flow model

Figure 2 illustrates one of the qualitative SD models developed in collaboration with the mental health trust. It uses the same stock and flow notation illustrated in Fig. 1. The model shown is focussed on the RIL teams. Several insights were gained in its construction. First, it was clear to all parties that that this was not a simple demand and treatment capacity problem. For example, a great deal of non-core work takes place due to monitoring of ‘discharged’ service users within social care. The fraction of service users who undergo monitoring is determined by the degree of trust between clinicians and social care teams. When trust is low, the fraction of service users monitored increases and vice versa. A similar soft issue can be found in the discharge of complex patients, i.e. those that require a combination of medication, management by GPs in the community, and social care input. In this case, there is a delay while GPs build confidence that it is appropriate for a patient to be discharged into their care. While this negotiation takes place, a patient still requires regular monitoring by a mental health clinician. Other systemic issues are also visible. For example, the long delays in beginning treatment lead to clinicians spending time contacting patients by phone *before* they were admitted. This all takes time and reinforces the delay cycle.

The results of the modelling were used to inform where interventions could be targeted. For example, a more detailed qualitative SD study to identify the trust issues between clinicians, social services, and general practitioners.

### OR as a tool for prospective evaluation

The second role of OR within implementation science is as a *prospective* evaluation tool. That is, to provide a formal assessment and appraisal of competing implementation options or choices before any actual implementation effort, commitment of resources or disinvestment takes place. Informally, this approach is often called *what-if analysis* [21]. A mathematical or computational model of a healthcare system is developed that predicts one or more measures of performance, for example, service waiting times, patients successfully treated, avoided mortality, or operating costs. The model can be set up to test and compare complex interventions to the status-quo. For example, decision makers may wish to compare the number of delayed transfers of care in a rehabilitation pathway before and after investment in services to prevent hospital admissions and disinvestment in rehabilitation in-patient beds. The approach has been applied widely in the areas outlined in the introduction to this article.

#### A case example—emergency medicine capacity planning

As a simple case example of prospective evaluation, consider the emergency department (ED) overcrowding problems faced by the United Kingdom’s (UK) National Health Service (NHS). The performance of NHS EDs is (very publically) monitored by recording the proportion of patients who can be seen and discharged from an ED within 4 hours of their arrival. The UK government has set a target that 95 % of service users must be processed in this time. In recent years, many NHS EDs have not achieved this benchmark. The reasons for this are complex and are not confined to the department [27] or even the hospital [15]. However, given the high public interest, many EDs are attempting to manage the demands placed on them by implementing initiatives to reduce waiting times and optimise their own processes.

Our case study took place at a large ‘underperforming’ hospital in the UK. The management team were divided in their view about how to reduce waiting times. One option was to implement a clinical decision-making unit (CDU). A CDU is a ward linked to the ED that provides more time for ED clinicians to make decisions about service users with complex needs. However, at times of high pressure, a CDU can also serve as *buffer capacity* between the ED and the main hospital. That is, a CDU provides space for service users at risk of breaching the 4-h target once admitted; service users are no longer at risk of breach. The question at hand was if a CDU were implemented, how many beds are required in order for the ED to achieve the 95 % benchmark?

##### Methods

**Fig. 3:**
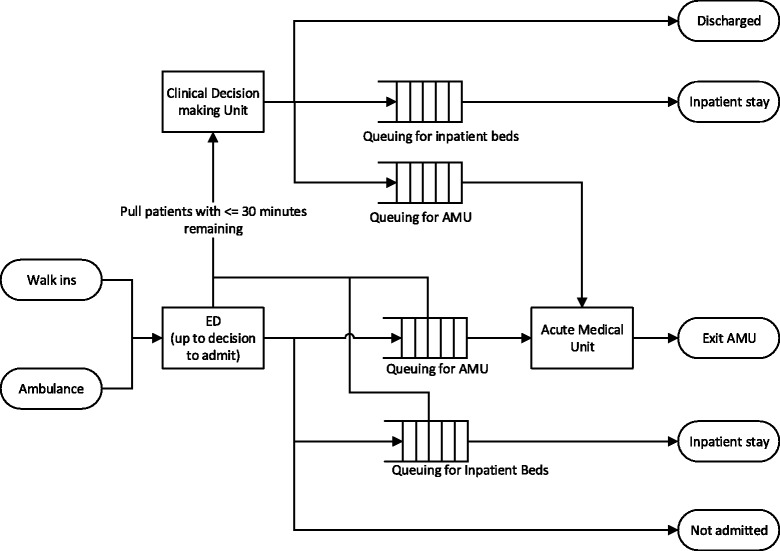
Emergency department and clinical decision-making unit model. Notation guide. *Rectangles* represent processes; *partitioned rectangles* represent queues; *ellipses* represent start and end points; *arrows* represent the direction of patient flow

Figure 3 illustrates the logic of a computer simulation model that was developed to evaluate the implementation of a CDU on ED waiting times. A computer simulation model is a simplified dynamic representation of the real system that in most cases is accompanied by an animation to help understanding. In this case, the simulation mimicked the flow of patients into an ED, their assessment, and treatment by clinicians and then flow out to different parts of the hospital or to leave the hospital entirely. The scope of the modelling included the hospital’s Acute Medical Unit (AMU) that admits medical patients from the ED. In Fig. 3, the rectangular boxes represent processes, for example, assessment and treatment in the ED. The partitioned rectangles represent queues, for example, patient waiting for admission to the AMU. The model was set up to only admit patients to the CDU who had been in ED longer than 3.5 h and only then if there was a free bed. Once a patient’s CDU stay was complete, they would continue on their hospital journey as normal, i.e. discharged home, admitted to the AMU or admitted to another in-patient ward.

In the model, the various departments and wards are conceptualised as stochastic queuing systems subject to constraints. This means that the variability we see in service user arrival and treatment rates (e.g. sudden bursts in arrivals combined with more complex and hence slower treatments) combined with limited cubicle and bed numbers results in queues. There are three reasons why prospective evaluation is appropriate for these systems. First, capacity planning for such complex systems based on average occupancy fails to take queuing into account and will substantially underestimate capacity requirements [28]. Second, the processing time, i.e. the time taken to transfer a patient to a ward and then to make a clinical decision, within a CDU is uncertain, although it is likely to be slower than the high pressure environment of the ED. Third, as the same ED and AMU clinicians must staff the CDU, the (negative or positive) impact on their respective processing times is uncertain.

The model developed was a discrete-event simulation [29] that mimics the variation in service user arrival and treatment rates in order to predict waiting times. The uncertainty in CDU processing time was treated as an unknown and varied in a *sensitivity analysis*. The limits of this analysis were chosen as 2 and 7 h on average, as these were observed in similar wards elsewhere.

##### Results

The model predicted that the number of CDU beds would need to be between 30 and 70 in order to achieve the ED target (for reference, the ED had 10 cubicles for minor cases and 18 cubicles for major cases). This result illustrated that even if a decision was made in 2 h on average with no negative effect on ED or AMU processing time, the CDU would need to be at least the same size as the ED overall. It also highlighted that the CDU impact on ED performance was highly sensitive to processing time.

The benefit of evaluating the CDU implementation upfront was that it ruled the CDU out as a feasible intervention before any substantial resource had been mobilised to implement it. The hospital could not safely staff a 30-bedded CDU or indeed provide space for that size of ward. As such, the modelling helped the management team abandon their CDU plan and consider alternative solutions with minimal cost and no disruption to the service.

### OR as a tool for strategic reconfiguration

The previous section described an implementation science approach to evaluate a small number of competing options at an operational level. In some instances, particularly in healthcare logistics and estate planning, a more strategic view of a system is needed to shortlist or choose options for reconfiguration. In such implementation problems, there may be a large number of options reaching into the hundreds, if not hundreds of thousands of competing alternatives. To analyse these problems, mathematical and computational optimization techniques are required. For example, if a provider of sexual health services wanted to consolidate community clinics from 50 to 20 and there are 100 candidate locations, then there are in the order of 10^20^ configurations to consider. OR’s implementation science role is to provide tools that identify options that help meet a strategic objective. For example, this might be maintaining equitable patient access to services across different demographics groups or modes of transportation while increasing service quality and reducing cost.

#### A case example—where should TIA outpatient clinics be located?

As a simple exemplar, consider a rural region in the UK that provided a 7-day transient ischemic attack (TIA) service through outpatient clinics in the community. Clinics ran at five locations but with only one location open per day. Magnetic resonance imaging (MRI) was available at three locations. Service users attending clinics without imaging but who require access to an MRI make an additional journey to the closest location with imaging capacity.

Service users are booked into clinic appointments across the week as they are referred to the TIA service by their diagnosing clinician, typically the patients local GP or an attending emergency department physician. The diagnosing clinician risk stratifies service users as high or low risk of a major stroke. High-risk service users require to be seen within 24 h of symptom onset and low-risk patients within 7 days [30].

The healthcare providers had concerns that splitting the clinics across five sites increased the variation in care received by service users and wished to consolidate to one to three clinic locations. Hence, there were two complicating factors when assessing equitable access: how many locations and which ones. There were also concerns that one location—clinic X—on the coast of the region was extremely difficult for high-risk TIAs to reach on the same day as diagnosis. There would also be political implications for any closure at clinic X. In total, there were 25 combinations of clinics for the providers to consider for both the low- and high-risk TIA groups, i.e. 50 options to review.

##### Methods

A discrete-choice facility location model was developed to evaluate the consequences of different TIA clinic configurations and inform the decision-making process for the reconfiguration of the service. Location analysis is a specialised branch of combinatorial optimisation and involves solving for the optimal placement of a set of facilities in a region in order to minimise or maximise a measure of performance such transportation costs, travel time, or population coverage [31]. In this, case an analysis was conducted separately for high-risk and low-risk TIAs. The analysis of high-risk TIAs aimed to minimise the maximum travel time of a service user from their home location to the closest clinic (as these service users must be seen the same day). The low-risk analysis minimised the weighted average travel time to their closest clinic. The weighted average measure allows for locations with the highest level of demand to have the greatest impact on results, diminishing the impact of outlying points. In general, if there are *n* demand locations and on a given day the travel time *x* from locations *i* to the nearest clinic, then the weighted average travel $\overline{x}$ time is given by the simple formula depicted in Eq. (1). Table 1 illustrates the use of the equation with two fictional locations. For each location, the number of patients who travel and the travel time for patients to a hospital is given. In the table, the weighted average is compared to the more familiar mean average.

1 $$ \overline{x}=\frac{{\displaystyle {\sum}_{i=1}^n}{x}_i{w}_i}{{\displaystyle {\sum}_{i=1}^n}{w}_i} $$

**Table 1:** Difference between weighted and unweighted averages

Location (*i*)	Patients (*w*_*i*_)	Travel time (*x*_*i*_; minutes)
1	1	30
2	5	10
Calculations		
Average travel time		$\frac{30+10}{2}=20\ \min$
Weighted average travel time		$\frac{\left(1\times 30\right)+\left(5\times 10\right)}{1+5}=13.3\ \min$

##### Results

The model demonstrated that clinics most central to the region were all good choices to provide equitable patient access. A three-clinic solution provided the most equitable solution for service users. The problematic clinic *X* on the coast of the region was not included in an optimal configuration; however, it could be included in a three-clinic solution without substantial effect on travel times if scheduled infrequently. This latter result allowed the decision makers to move on from the strategic debate about location and focus on the more detailed implementation issues of scheduling and capacity planning for clinics. This was again addressed upfront using a computer simulation study to evaluate a small number of competing options for scheduling the clinics.

### Lessons for implementation science

Each of the three roles emphasises the use of OR to conduct implementation science upfront before any action to alter a care pathway or service has been taken. Many OR scholars argue that the benefit of constructing a model upfront is that it forces decision makers to move from a world of imprecise language to a world of a precise language (sometimes referred to as a common language [32]) and ultimately develop a shared understanding of the problem; although as I will argue later, there is very limited empirical evidence supporting this proposition. Such a shared understanding increases the likelihood if implementation will actually go ahead and importantly if it will be sustained or normalised.

It is important to emphasise that the three case studies illustrate the simpler end of what can be achieved in using OR for upfront implementation science. This is partly a stylistic choice in order to aid reader understanding, for example, many optimisation problems are hugely complex, but also because in my experience simpler models tend to be accepted and used more in healthcare. Simpler models also need less input data and hence can be built and run quickly.

Along with the three case studies, OR is in general grounded in the use of models to improve upfront decision-making in complex implementation problems. Although there is a significant overlap between OR and implementation research, there are differences. For example, OR would not provide the rich contextual information collected in a process evaluation.

### Implementation science challenges for OR

Implementation science poses a number of challenges for OR. I propose that these lie at three levels: disciplinary, organisational, and interventional. Table 2 summarises these key challenges.

**Table 2:** Implementation science challenges for OR

Level	Challenge
Disciplinary	• Evidence of impact and effectiveness • Understanding of practice • Involvement of service users in research
Organisational	• Awareness of operational research • Engagement of decision and policymakers
Interventional	• Mutual understanding between stakeholders and researchers • Parameterisation of models

#### Challenges at a disciplinary level

This article describes three roles for OR within implementation science. An irony is that OR interventions themselves are poorly understood with barely any published evaluation of practice or impact [33–36]. Limited examples can be found in Monks et al. [37], Pagel et al. [38], and Brailsford et al. [39]. The explanation for this can be found at a disciplinary level. That is, academic OR is predominately driven and rewarded by the development of theory for modelling methodology as opposed to understanding interventions and the issues they raise for practice. As such, a discipline that promotes the use of evidence for decision-making in healthcare cannot confidently answer the question *does OR in health work*? I am regularly challenged on this point by healthcare professionals.

A second disciplinary challenge is to systematically involve service users in the co-design of OR interventions. To date, evidence of service user involvement is limited (see Walsh and Holstick [40] for an example). There is also confusion between service users framed as research participants (typically treated as a data source to parameterise models with behavioural assumptions) and co-designers of research objectives and methods, although there has been an effort to clarify the important difference [41].

#### Challenges at the organisational level

The three roles of OR outlined above are widely applicable across healthcare implementation problems. However, before OR can be used within practice, users of the research, in this case, healthcare managers, clinicians and service users, must be aware of the approaches. This is currently a substantial barrier to a wide scale adoption in health services [42–44] and stands in stark contrast to domains such as manufacturing and defence where it is used frequently to generate evidence before action [45]. The implication of low awareness of OR in health is that it is often difficult to engage senior decision makers in the complex operational and logistical problems that matter the most for service users.

#### Challenges at an interventional level

Fifty years ago, Churchman and Schainblatt [46] wrote about a ‘dialectic of implementation’ in the journal *management science.* In this paper, the two authors advocated that a position of *mutual understanding* between a researcher and manager was necessary in order to implement results of a study. That is, the researcher must understand the manager’s position, values, and implementation problem in order to tackle the correct problem in the right way. The manager must understand the method that the researcher has applied, at least at the conceptual level, in order to scrutinise, challenge, and implement results. The concept of mutual understanding is an elegant one, but in practice, achieving it is a challenge for both sides. As a simple example from a researcher perspective, it is difficult to assess if the users of a model understand why a model is producing certain results [42]. That is, do users understand how the model works or are they simply accepting the results based on some heuristic, such as ‘these are the results I want’ or ‘I trust the person telling me the results’? Given the disciplinary challenge outlined above, to date, there is limited validated guidance about how to manage such complex interventions within OR.

The computer software used in the three case studies have been available for considerable time, but appropriate data to parameterise the quantitative models used to illustrate the second and third roles are potentially not collected routinely. All models require data from the system studied. The TIA clinic study had relatively low requirements: individual service user-level data detailing date of clinic attendance, clinic attended, the risk classification of patient, and a home location of the patient—much of which is collected routinely by a health system for financial reporting purposes. Simulation modelling studies such as that described in the emergency department case study have high data requirements, including fine-grained timings of processes such as triaging and doctor assessment. It is unlikely such data are collected routinely as they have no use in financial reporting.

### An agenda for OR in implementation science

Given the organisational, interventional, and disciplinary issues outlined in the ‘Implementation science challenges for OR’ section, I propose the following agenda for OR within implementation science.

#### Priority 1: creating the evidence base

At the forefront of the research agenda is the need to evaluate the impact of OR on complex interventions. The focus here should be on the consumers of research as opposed to the modellers and the process they follow [47, 48]. There is a need to understand how stakeholders make sense of an OR intervention and how the results of studies are used to assist decision-making. Recent research offers some promise in progressing this aim. PartiSim [49] is a participative modelling framework that aims to involve stakeholders in structured workshops throughout a simulation study. Structured frameworks like PartiSim provide an opportunity to study the user side of OR more efficiently, as the modelling steps are known upfront. Another area showing promise is the recent emergence of Behavioural OR [50]. One of the core aims of Behavioural OR is to analyse and understand the practice and impact of OR on context (e.g. [51–53]).

#### Priority 2: raising demand and the liberation of OR

Much of the challenge in the use of OR as an implementation science technique that I outline is rooted in the lack of organisational awareness and experience of the approach. But what if this challenge were to be resolved? To examine this further, consider a counterfactual world where all health service users, managers, and clinicians are well versed in the three implementation science roles of OR and all have free access to a substantial evidence base detailing the efficacy of the approach. In this world, where OR is an accepted implementation science approach, the constraint has now moved from demand to supply of modelling services. Current supply is predominately provided by the (relatively) small specialist consultancy and research communities. There is a great need to *liberate* OR from its roots as the tool of the ‘specialist’ and transfer knowledge to research users. Two initial efforts to achieve this priority include the Teaching Operational Research for Commissioning in Health (TORCH) in the UK [54] and the Research into Global Healthcare Tools (RIGHT) Project [55]. TORCH successfully developed a curriculum for teaching OR to commissioners, although it has yet to be implemented on a wide scale or evaluated. The RIGHT project developed a pilot web tool to enable healthcare providers select an appropriate OR approach to assist with an implementation problem. Both of these projects demonstrate preliminary efforts at liberating OR from the traditional paradigm of specialist delivery.

The liberation of OR has already taken place in some areas in the form of *Community OR*. The three case studies illustrated interventions where the collaboration puts the emphasis on a modeller to construct the model and provide results for the wider stakeholder group. Alternatively, service users could develop or make use of OR methods to analyse a problem themselves. Community OR changes the role of an operational researcher from a modeller to a facilitator in order to aid those from outside of OR to create appropriate systematic methodology to tackle important social and community-based issues. In a rare example of community OR in healthcare [40], two examples illustrate where service users take the lead. In the first example, users of mental health services used system methods to produce a problem structuring tool to evaluate the impact of service users on NHS decision-making. In the second example, service users developed and applied an idealised planning approach for the future structure of mental health services. These approaches are qualitative in nature but are systematic and in-line with an OR implementation science approach.

#### Priority 3: PPI education for OR modellers

The first two priorities listed might be considered long-term goals for the OR implementation science community. An immediate priority that is arguably achievable over the short term is Patient and Public Involvement (PPI) education for OR modellers. The co-design of healthcare models with decision makers is often held up as a critical success factor for modelling interventions [42]. For ethical and practical reasons, co-design of OR modelling interventions should also include service users [41]. Education need not be complicated and could at first be done through widely read OR magazines and a grass roots movement delivered through master degree courses.

## Conclusions

Operational research offers improvement scientists and individuals who work in complex health systems the opportunity to do more *upfront system thinking* about interventions and change. OR's upfront role within implementation science aims to answer questions such as where best to target interventions, will such an intervention work even under optimistic assumptions, which options out of many should we implement, and should we consider de-implementing part of a service in favour of investing elsewhere. As OR becomes more widely adopted as an implementation science technique, evaluation of the method through the lens of implementation science itself becomes more necessary in order to generate an evidence base about how to effectively conduct OR interventions. It is also necessary to liberate OR from its traditional roots as a specialist tool.

### Summary

Operational research (OR) is a mature discipline that has developed a significant volume of methodology to improve health services. OR offers implementation scientists the opportunity to do more upfront system thinking before committing resources and taking risks. OR has three roles within implementation science: structuring an implementation problem, upfront evaluation of implementation problems, and a tool for strategic reconfiguration of health services. Challenges facing OR as implementation science include limited evidence or evaluation of impact, limited service user involvement, a lack of managerial awareness, effective communication between research users and OR modellers, and availability of healthcare data. To progress the science, a focus is needed in three key areas: evaluation of OR interventions, transferring the knowledge of OR to health services, and educating OR modellers about the aims and benefits of service user involvement.

## Abbreviations

AMU, Acute Medical Unit; CDU, clinical decision-making unit; ED, emergency department; GP, general practitioner; MRI, magnetic resonance imaging; NHS, National Health Service; OR, operational research (UK)/operations research (US); PPI, Patient and Public Involvement; PPT, psychology and psychiatric talking therapies; RIGHT, Research into Global Healthcare Tools; RIL, recovering independent life; SD, system dynamics; TIA, transient ischemic attack; TORCH, Teaching Operational Research for Commissioning in Health
